# Analysis of oral microbial dysbiosis associated with early childhood caries

**DOI:** 10.1186/s12903-021-01543-x

**Published:** 2021-04-07

**Authors:** Hui Zheng, Tengfei Xie, Shaokai Li, Xiaotong Qiao, Youguang Lu, Yan Feng

**Affiliations:** 1 Stomatological Key Laboratory of Fujian College and University and Department of Orthodontics, School and Hospital of Stomatology, Fujian Medical University, Fuzhou, China; 2 Fujian Provincial Engineering Research Center of Oral Biomaterial and Department of Preventive Dentistry, School and Hospital of Stomatology, Fujian Medical University, Fuzhou, China; 3 School and Hospital of Stomatology, Fujian Medical University, Fuzhou, China; 4 Institute of Stomatology and Department of Preventive Dentistry, School and Hospital of Stomatology, Fujian Medical University, 246 Middle Yangqiao Road, Fuzhou, 350000 China; 5 Fujian Key Laboratory of Oral Diseases and Department of Preventive Dentistry, School and Hospital of Stomatology, Fujian Medical University, 246 Middle Yangqiao Road, Fuzhou, 350000 China

**Keywords:** Early childhood caries, Oral microbiota, Microbial dysbiosis, *16S rRNA* gene, *Streptococcus mutans*

## Abstract

**Background:**

"Core microbes" play a key role in the development of caries and lead to microbial disorders. Our goal was to detect the core microbes associated with the microbiota imbalance in early childhood caries (ECC).

**Methods:**

Fifteen caries-free children and fifteen high-caries (DMFT ≥ 10) children aged 4–6 years old were recruited according to the diagnostic criteria of caries suggested by the WHO. The *16S rRNA* genes from samples of plaque in saliva were amplified by PCR, and the PCR products were sequenced by the Illumina Miseq platform. The sequencing results were analyzed by professional software to determine the composition and structure of the saliva microorganisms.

**Results:**

There were statistically significant differences between the groups regarding the relative abundance of *Streptococcus mutans* (Wilcoxon rank-sum test, *P* < 0.05). No significant difference was found between the groups regarding other species or functional genes.

**Conclusion:**

*S. mutans*, together with other pathogens, may play a prominent role and act as "core microbes" in the occurrence and development of early childhood caries.

## Background

Early Childhood Caries (ECC) is defined as one or more decayed (non-cavitated or cavitated lesions), missing or filled (due to caries) surfaces presenting in any deciduous tooth of a child under six years old [1]. The high prevalence of ECC among these young children around the world has a significant impact on children's health and social costs. However, surveys have made it clear that, except for the prevalence of ECC, it is largely untreated in children under the age of three [2]. The caries rate of deciduous teeth in China is 71.9% in 5-year-old children, and the DMFT is 4.24. The significant caries index (SIC) of these children was found to be 9.61 [3]. Severe early childhood caries can cause masticatory dysfunction, malocclusion, jaw dysplasia, temporomandibular joint disorder, facial aesthetic problems and abnormal pronunciation, which will have unpredictable impacts on children's lives. Therefore, the tasks of prevention and treatment of deciduous teeth caries are still arduous, and more research on the etiology of early childhood caries is urgently required.

According to the quadruple factors theory of caries, bacteria, food, host and time are all necessary conditions for caries occurrence. Dental caries have been defined as microbial dysbiosis [4]. Bacterial factors are important factors in caries etiology and bacteria depend on biofilm to cause caries. For the microbial factors of dental caries, it is generally accepted that microorganisms ferment carbohydrates in dental plaque to produce acid, which leads to the breakdown of the balance between demineralization and remineralization of enamel [5].

At present, *Streptococcus mutans, Lactobacillus spp., Actinomyces* and other microorganisms are associated with caries, which is also related to the individual's behaviors and caries resistance. However, no microorganism has been found to conform to Koch's postulates of pathogenic bacteria that can prove that caries is an infectious disease caused by a single species. Currently, based on molecular biology methods such as denaturing gel gradient electrophoresis (DGGE) [6], human oral microbe identification microarray (HOMIM) [7] and *16S rRNA* gene sequencing [8], no caries-specific bacteria have been found, but different caries-related bacteria species have been observed in different studies.

Research on the microbial etiology of dental caries has not been not well developed, partly due to the complexity of the etiology of dental caries itself. It is estimated that dental plaque contains approximately 700 organic combinations and interacting bacterial communities, which greatly increases the difficulty of the research on the pathogenic bacteria of dental caries. The specific plaque hypothesis argues that specific bacterial species in the plaque, such as *S. mutans,* are the key pathogenic factors of caries, while according to the nonspecific plaque hypothesis, the acidic secretion activity of the entire microbial plaque community is increased, leading to enamel demineralization [9].

Regarding the relationship between dental caries and microorganisms, an increasing number of scholars agree with the ecological plaque hypothesis [10, 11], which postulates that the occurrence of dental caries is the result not of the activity of a specific microorganism, but rather of external factors. The destruction of the microecological balance in plaque biofilm and the disorder of the overall microbial community structure result in the occurrence and development of dental caries. Therefore, it is necessary to study the composition and structure of the entire microbial community and to obtain comprehensive information on the microorganisms in the plaque biofilm. With the development of metagenomic technology and DNA sequencing technology, it is now possible to directly analyze the species composition and functional genes in the community by obtaining the microbial genome of the whole community [12].

Studies on the microbiota associated with early childhood caries suggest that the microecological imbalance leading to caries in children may be caused by multiple microorganisms, which differs from the suggestion that the pathogenic microorganisms, obtained through traditional culture methods, are *S. mutans, Lactobacillus spp.* and *Actinomyces*. Based on the existing literature on caries disease and early childhood caries and on some clinical studies of caries-susceptible children, we proposed the hypothesis that there are some "core microbes" associated with caries in dental plaque and that these "core microbes" play a key role in the development of the occurrence of dental decay and determine the microbial disorders.

In this study, in order to analyze the salivary microbial composition and structure, we compared the plaque samples loaded in saliva from children with no caries and children with ECC using *16S rRNA* gene sequencing technology. Our aim was to detect pathogenic bacteria that were related to the microbial dysbiosis of ECC, providing a theoretical basis for caries prevention and therapy.

## Methods

### Participants recruiting

Fifteen caries-free children were recruited in control group (group C) and fifteen high-caries children in disease group (group D) for this project. Their basic information (sex, age, feeding habits and oral hygiene) and their caries situation were recorded. Informed consent was obtained from the children's parents for this research. The inclusion criteria for the children with high caries (DMFT ≥ 10) were based on the diagnostic criteria of caries formulated by the WHO [Oral Health Surveys: Basic Methods (fourth edition)]. The inclusion criteria were as follows: (1) Evidence of caries present (a lesion in a pit or fissure or on a smooth tooth surface, an unmistakable cavity, undermined enamel, detectably softened floor or wall, a tooth with a temporary filling or one that was sealed but also decayed, including white spot lesions and dental caries losses); (2) Children were between 4 and 6 years old, with primary teeth that have erupted completely and permanent teeth that have not yet erupted.

The exclusion criteria for all children were as follows: (1) there was evidence of other oral diseases, family hereditary diseases, systemic diseases, long-term medication history or use of antibiotics in the past three months. (2) Those who with first permanent molars and/or permanent incisors erupted. Fifteen children classified as healthy (dmft = 0) were included in the control group according to the exclusion criteria.

The project was approved by the Ethics Committee of the Stomatological Hospital Affiliated with Fujian Medical University (Committee’s reference number: 2017–30).

### Collection of plaque and salivary mixture specimens

The dental plaque of the children was sampled 0.5–3 h after breakfast without brushing teeth. Oral examination was performed by an experienced dentist in the form of a visual examination combined with exploratory probing. When sampling, the researchers let the children brush their teeth without gargling by an aseptic toothbrush and spit their saliva into an aseptic paper cup. The cup contents were then poured into an aseptic 50 mL centrifugal tube, transferred to the laboratory in an ice box and stored in a freezer at—80 °C.

### Sample processing and DNA extraction

The plaque and saliva mixtures were thawed in the centrifugal tube. After thawing, the saliva samples were centrifuged at 12,000 rpm for 10 min and the diposit was used for DNA extraction. A bacterial genomic DNA extraction kit (Omega Bio-Tek, Inc. USA) was used to extract the bacterial genomic DNA from the samples. The extraction procedure was carried out according to the instructions of the kit.

### DNA library construction and sequencing

The DNA solution of all samples was examined by 1% agarose gel electrophoresis. A Qubit fluorometer was used for DNA quantitation and quality inspections. The DNA samples were stored at − 20 °C after extraction until further processing. Universal primers for the bacterial *16S rRNA* gene were used to amplify the bacterial *16S rRNA* gene v3-v4 variable regions (338F: 5′-ACTCCTACGGGAGGCAGCAG-3′, 806R: 5′-GACTACHVGGGTWTCTAAT-3′). The primers contained a tag sequence of 8 nt (Barcode) for distinguishing each sample. Each sample underwent three PCRs, with each 25 μL PCR containing 10 μL Pyrobest buffer, 2 μL dNTPs (2.5 mM), 1 μL primer (10 μM), 0.4 U Pyrobest DNA polymerase and 15 ng genomic DNA (template DNA). The PCR conditions used included denaturation at 95 °C for 5 min, followed by 25 cycles of denaturation at 95 °C for 30 s, annealing at 55 °C for 30 s, extension at 72 °C for 30 s, and a final extension at 72 °C for 10 min. The PCR products were used for subsequent Miseq sequencing (Illumina Inc., CA, USA). The PCR products were electrophoresed in a 2% agarose gel, followed by use of the AxyPrep DNA Gel Extraction Kit (Axygen Biosciences, CA, USA) according to the product manuals. The 16S rDNA library with barcodes was acquired after extension. QuantiFluor™-ST (Promega, Madison, USA) was used to quantify the DNA. The DNA of each sample was mixed in equal amounts, and the DNAs were sequenced on the Illumina Miseq platform according to the standard operating procedure (2 × 300 bp paired-end). Quality control of the DNA, construction of the *16S rRNA* gene fragment DNA library, and DNA sequencing were all performed by the sequencing company (Allwegene, Beijing, China).

### Data analysis of the sequencing results

1. The original data (*.fastq file) were split according to the specific label sequence (barcode) of the sample, and the FastQC software was used to check whether the quality and quantity of the original data met the basic requirements of the analysis;
2. A trimmingReads.Pl script in ngs-qc-toolkits was used to remove the 30 bp sequence at the 3′ end of read2 and to retain the high-quality 270 bp at the 5′ end;
3. An IlluQC.Pl script in ngs-qc-toolkits was used to screen out sequences whose quality Q20 was less than 90% (parameter: − l 90-s 20);
4. Paired read1 and read2 were processed in the Mothur pipeline and first merged using Mothur > make.contigs, with default parameters. Merged sequences with a length > 489 bp were discarded. Follow-up analysis was performed according to the analytical procedures of Mothur. The SILVA database was selected for alignment and taxonomic annotation. Chimera removal was not performed, and 80% for the taxonomic annotation cutoff was selected. A cluster.split method was used to cluster sequences into OTUs. This resulted ultimately in a *. biom file;
5. QIIME 1.8 was used to further analyze the *. biom file. We removed the low abundance OTUs in the *.biom file (total count 2 in all samples) and generated the relative abundance table from the phylum to the genus level;
6. In QIIME 1.8, a certain sequence number (n = 18,600) was randomly selected to calculate the alpha diversity of all samples (Chao1 index and Shannon index);
7. The Bray–Curtis distance was calculated between samples (Qiime > beta_diversity.Py-m bray_curtis). At the same time, based on the Bray–Curtis distance matrix, the PCoA visualization of the distance between the samples was carried out with R programming language.

### Specific details on the real-time PCR assays

Universal primers targeted to total bacteria and specific primers targeted to *S. mutans, Lactobacillus spp.* and *Streptococcus sobrinus* were chosen to determine the bacterial load by real-time PCR (qPCR). Functional genes, including the *ldh* gene and the *F-ATPase* gene, were also quantified by real-time PCR (qPCR). The primers are listed in Table 1. The samples were tested in triplicate. Each PCR was performed in a total volume of 20 μl, which contained 10 μl of Fast Power SYBR green PCR Master Mix, 500 nM of each primer pair, 1 μl DNA template and PCR water. The thermocycler conditions included a denaturation step of 95 °C (2 min) and 40 cycles of 95 °C (15 s) and 60 °C (1 min). All samples were amplified in triplicate, and the mean value of the targeted molecule was used for analysis. We used the total (universal) bacterial *16S rRNA* gene as the reference gene to calculate the relative abundance of the specific *16S rRNA* genes of the target bacteria and the relative expression level of the functional genes. For total bacteria determination, the DNA templates were diluted 100-fold, and for the functional gene determination, the templates were diluted tenfold. The undiluted DNA solutions were used as the DNA templates in each PCR. The relative abundance of the specific taxa was simply defined as the relative abundance of the detected gene sequences mapped to the reference genome. The relative abundance of specific taxa or functional genes was simply defined as the ratio of DNA fragments detected to the total bacteria detected via qPCR. The Ct values of the target genes (Ct target) minus the Ct values of the total bacterial gene (Ct total-bacteria) were used to determine the ΔCt values. The values of 2^-ΔCt were defined as the relative abundance of the specific taxa or genes.

**Table 1:** Primers used in qPCR

	Forward primer	Reverse primer	Amplicon size (bp)
Total bacteria	5′-TCCTACGGGAGGCAGCAGT -3′	5′-GGACTACCAGGGTATCTAATCCTGTT-3′	466
*S. mutans*	5′-GCCTACAGCTCAGAGATGCTATTCT-3′	5′-GCCATACACCACTCATGAATTGA-3′	98
*Lactobacillus spp.*	5′-TGGAAACAGGTGCTAATACCG-3′	5′-GTCCATTGTGGAAGATTCCC-3′	231
*S. Sobrinus*	5′-TTCAAAGCCAAGACCAAGCTAGT-3′	5′-CCAGCCTGAGATTCAGCTTGT-3′	88
*ldh* gene	5′-CTTGATACTGCTCGTTTCCGTC-3′	5′-GAGTCACCATGTTCACCCAT-3′	93
*F-ATPase* gene	5′-CGGATGCGTGTTGCTCTTACTG-3′	5-GGCTGATAACCAACGGCTGATG-3′	167

### Statistical analysis

Alpha diversity among the different groups was compared by an independent sample t test. The comparison of the relative abundance of the bacterial species used the Wilcoxon rank-sum test. Multiple comparisons were compared by the Bonferroni test correction. The data in this paper are expressed as the mean + standard deviation. There was statistical significance if the p value was less than 0.05.

## Results

After quality inspection, trimming, filtering, splicing and refiltering of the raw sequencing data, 4 samples were excluded, due to the number of sequences in a single sample of less than 5000 (including 2 samples from the caries-free group and 2 samples from the disease/ECC group). Thirteen samples from the control group (group C) and 13 samples from the disease group (group D) were obtained. A total of 683,709 sequences were obtained, with sample C27 showing the largest number (38,055) of sequences and sample D33 showing the smallest number (18,657) of sequences, with a median sequence number of 25,816.

### Alpha diversity analysis based on OTUs

**Fig. 1:**
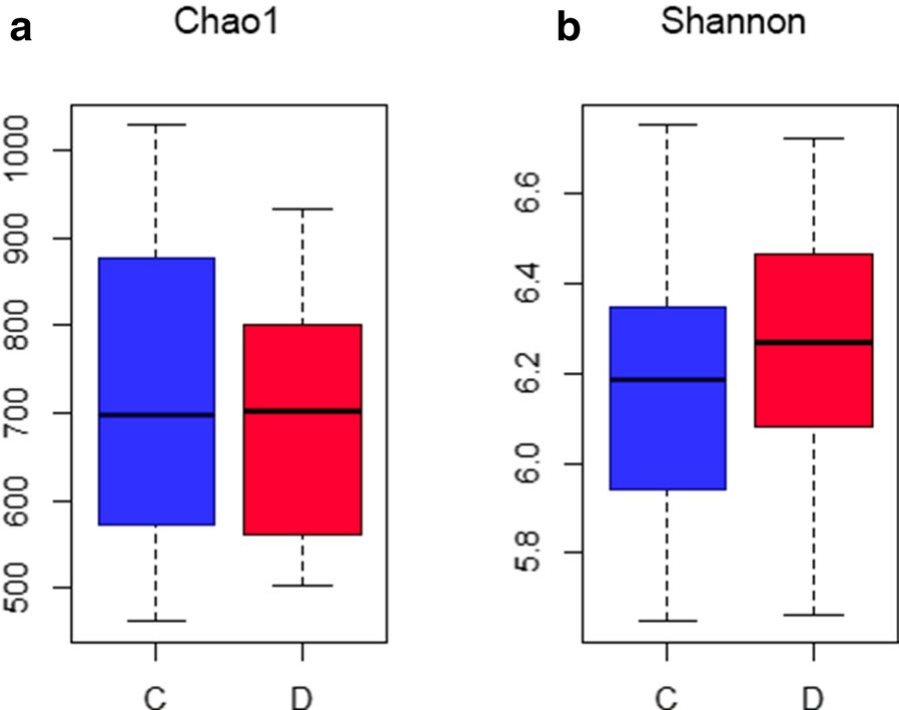
Comparison of microbial richness and diversity *C* control group/no caries group; *D* disease group/ECC group

With the minimum sequences, sample D33 (18,657 sequences) was defined as the threshold for sequence re-sampling, 18,600 sequences were randomly selected from all samples (10 times for each sample) to calculate the alpha diversity. The Chao1 index (indicating microbiota abundance) and Shannon index (indicating microbiota diversity) of group C and group D were not significantly different (*P* > 0.05, Fig. 1).

### Bacterial community structure characteristics based on the Bray–Curtis distance

**Fig. 2:**
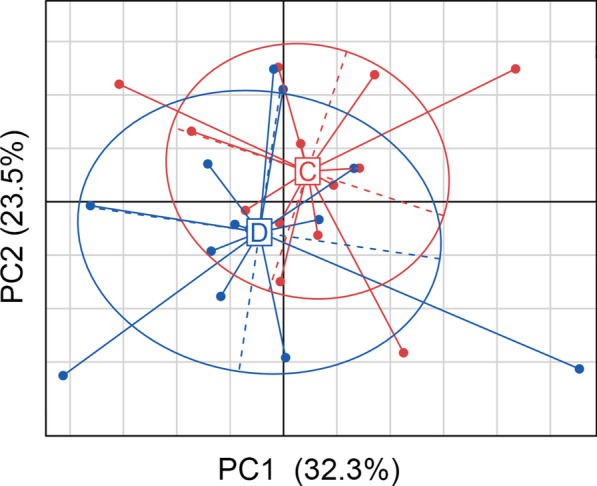
Comparison of microbial community structure between no caries group (C) and disease group/ECC group (D). PCoA visualization was performed according to Bray–Curtis distance maritrix between samples based on the relative abundance table of all taxonomic levels

We converted the OTU relative abundance table into a relative abundance table of taxonomic level from phylum to genus. We calculated the Bray–Curtis distance between the samples based on the relative abundance table of all taxonomic levels, after which PCoA visualization was performed (Fig. 2). The samples from group C and group D could not be separated significantly in principal composition 1 or principal component 2. We further analyzed whether there were significant differences in the taxonomic composition between the two groups based on the Bray–Curtis distance matrix, and no significant differences were found (permutation 999 times, p value of adonis was 0.272, p value of anosim was 0.368). According to this result, we found that we cannot distinguish the children with caries from the children without caries based on the bacterial community structure above the species level.

### Analysis of bacterial composition of the control group and caries group

**Fig. 3:**
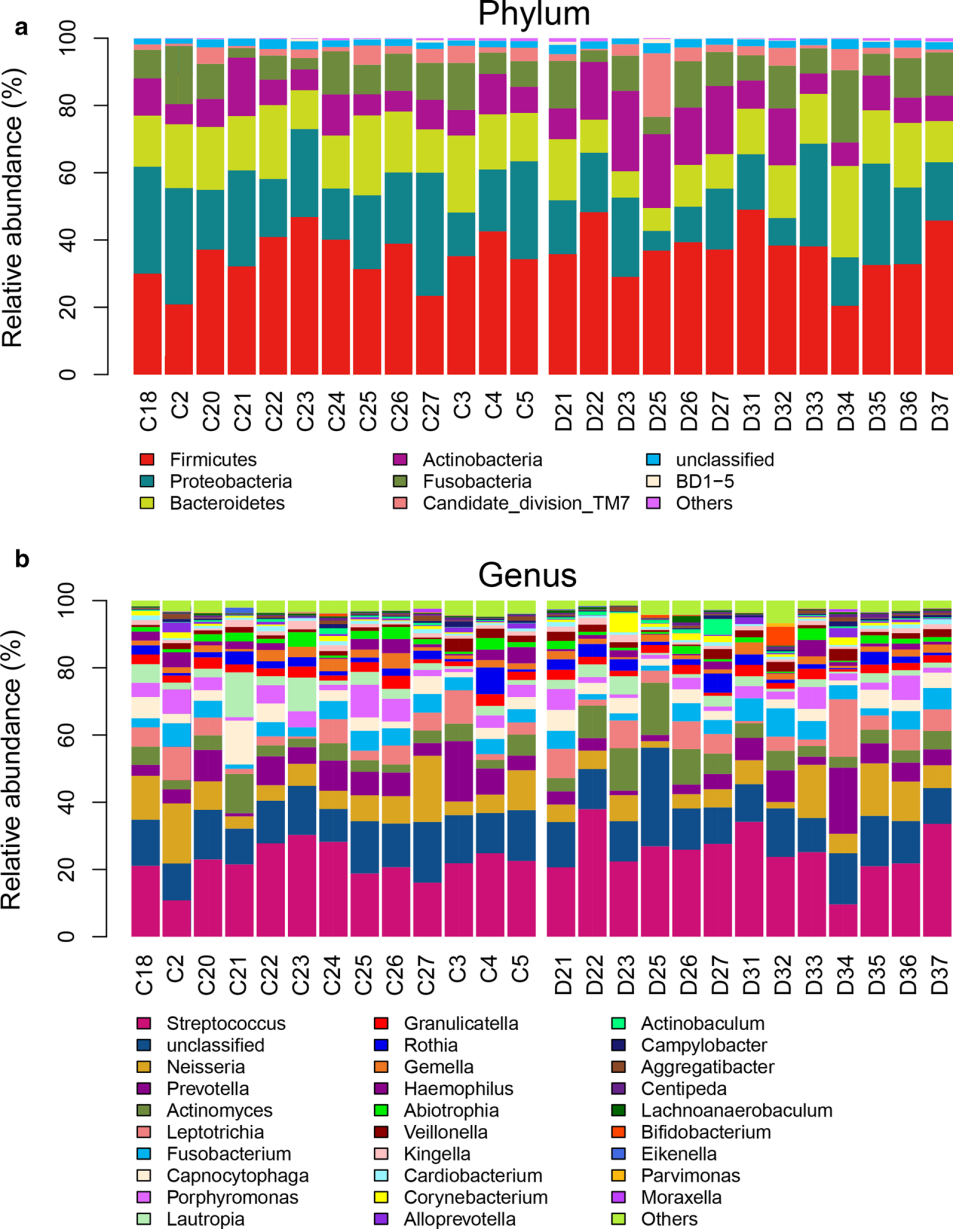
The predominant phylum and genus of the all samples

We generated the relative abundance table of taxa at each level (phylum, class, order, family and genus) from the OTU relative abundance table, and the relative abundance of taxa at the genus level is shown in Fig. 3. *Streptococcus* (23.8%), *Neisseria* (8.1%), *Prevotella* (6.2%), *Actinomyces* (5.6%) and *Leptotrichia* (5.4%) were found to be of the highest relative abundance. Based on our detection level and the current information of the *16S rRNA* gene database, no specific taxa were found in the ECC group in this study. The relative abundance of the taxa at the different levels was compared. After multiple comparison corrections (*P* < 0.05 as the boundary), no taxa with significant differences were found at any taxa levels. However, we found some genera that may be associated with caries, but those differentially present genera were all of low abundance (Table 2).

**Table 2:** The difference of relative abundance of genera between control group and caries group

Genus	Wilcoxon rank-sum test *P* value	C group (health)		D group (ECC)	
		Mean value	SD	Mean value	SD
*Bifidobacterium*	0.001235	0.000834	0.002135	0.005777	0.015335
*Proteiniphilum*	0.010454	0.000321	0.000407	9.35E−05	0.000267
*Shuttleworthia*	0.025254	5.17E−05	6.01E−05	0.00027	0.000528
*Legionella*	0.037349	0	0	2.04E−05	4.11E−05
*Hespellia*	0.037349	2.15E−05	4.97E−05	0	0
*Anaerosporobacter*	0.037481	0.000307	0.000305	0.000119	0.000164
*Dysgonomonas*	0.041659	0.000135	0.000179	2.42E−05	5.95E−05
*Caldicoprobacter*	0.043438	8.91E−05	9.75E−05	2.86E−05	7.6E−05

*SD* standard deviation

### Description of qPCR results

**Fig. 4:**
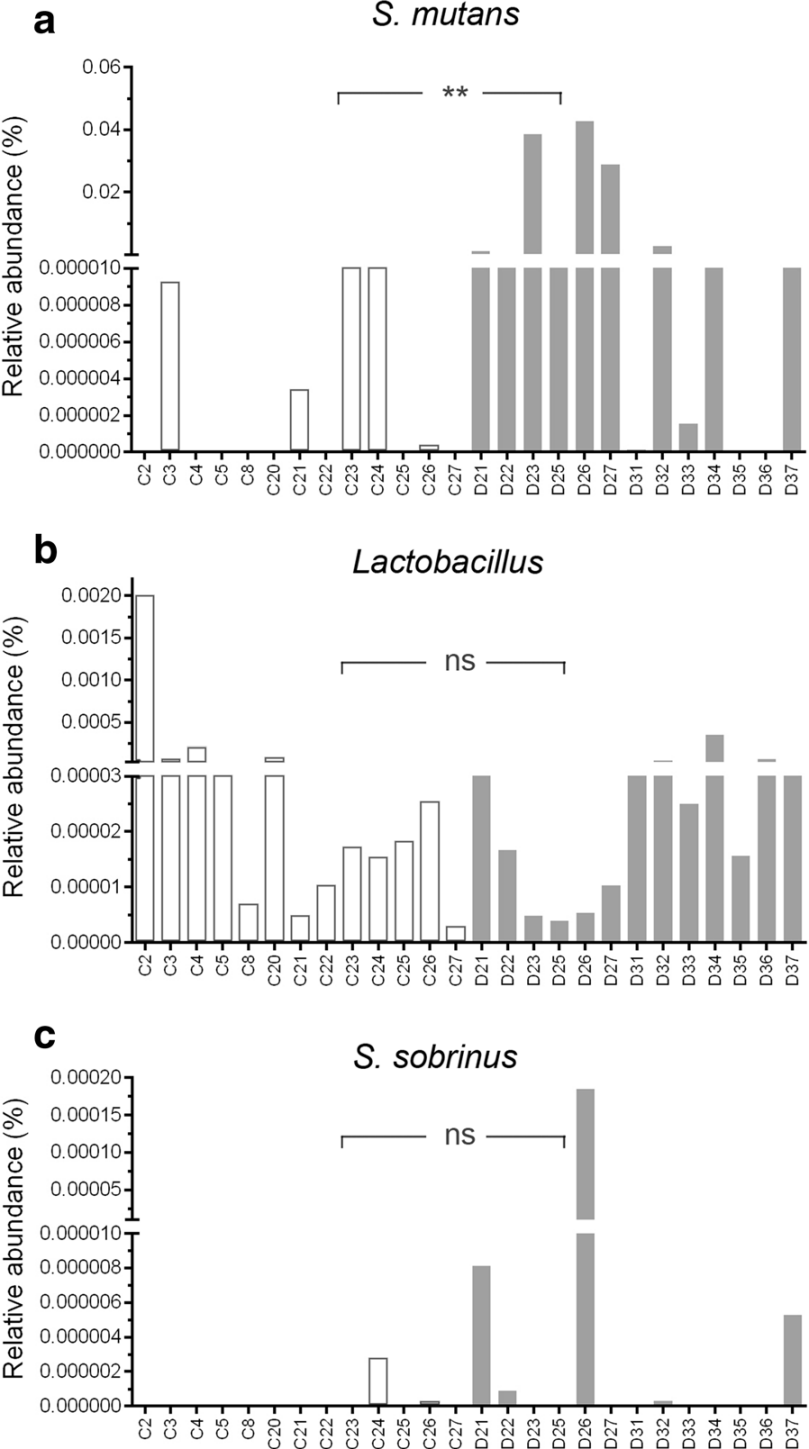
Comparison of the relative abundance of *S. mutan*, *Lactobacillu* and *S. Sobrinus.* The relative abundance were calculated based on qPCR results. *S.muans* differing in terms of relative abundance between no caries group (C) and disease group/ECC group (D) (Wilcoxon rank-sum test, *P* < 0.05). **: *P* < 0.05, *ns* not significant

**Fig. 5:**
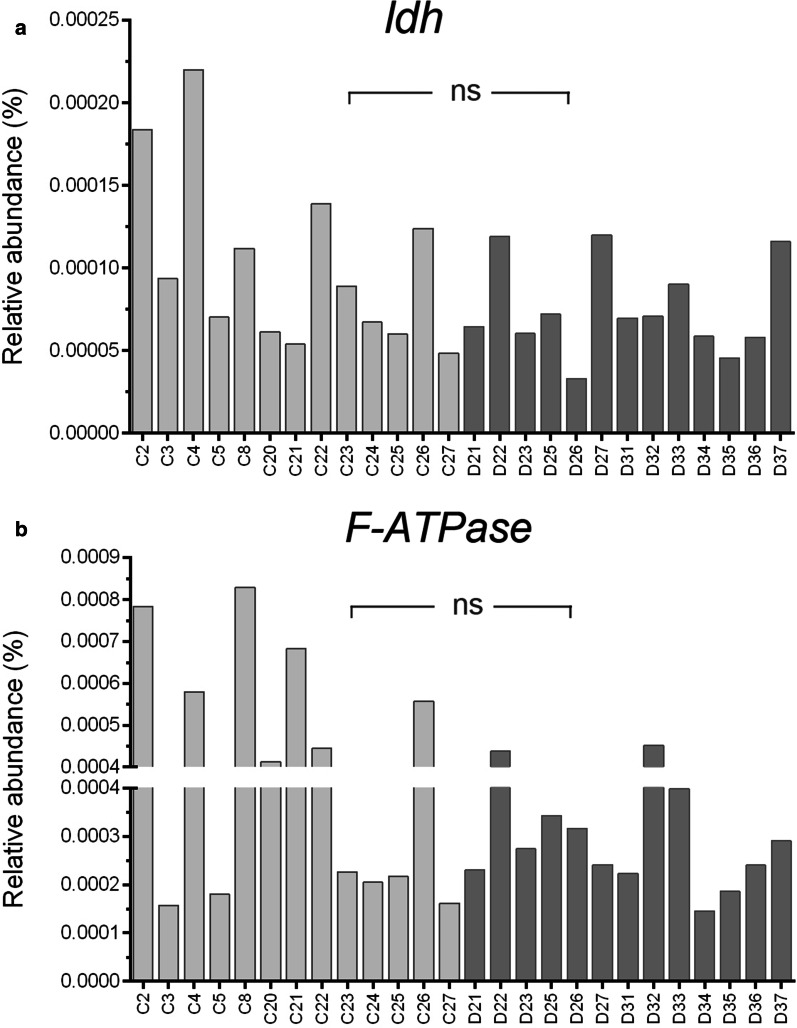
Comparison of the relative abundance of functional genes. The relative abundance were calculated based on qPCR results, *ns* not significant

The relative abundance of bacteria and expression of genes were calculated with the total number of bacteria as the internal parameter. There were statistically significant differences between the groups regarding the relative abundance of *S. mutans* (Wilcoxon rank-sum test, *P* < 0.05). No significant difference was found between the groups regarding other species (Fig. 4). No significant differences were found in functional genes (Fig. 5). The *S. mutans* of group C was maintained at a low level, with an average value of 3.78 × 10^–6^ (with a range from 1.46 × 10^–10^ to 2.24 × 10^–5^), while the relative abundance of *S. mutans* in group D varied, with a mean value of 8.89 × 10^–3^ (with a range from 2.72 × 10^–10^ to 4.30 × 10^–2^).

## Discussion

For decades, acid-producing bacteria, represented by *S. mutans,* have been considered to be the main pathogenic bacteria of caries. The characteristics of extracellular polysaccharide production, acid production and acid tolerance support the role of *S. mutans* as a potential cariogenic bacteria, and most preventive measures and risk assessment methods for caries have been targeted at *S. mutans* [13]. Recently, a metagenomic method was used to study caries disease-related microbes, which found that *S. mutans* in plaque biofilm was not the dominant bacteria and that its proportion in the whole microbiota was very low. In some studies, *S. mutans* often cannot be detected, even by using metagenomic detection methods [14, 15]. We sequenced saliva samples from two groups of children, and there was no differential presence between the two groups in *S. mutans* found in the saliva. This does not mean that *S. mutans* is unimportant. The possible explanation is that *S. mutans* plays a role in the initial stage of caries formation (demineralization period) and, at a low abundance, also may play a role as a key pathogenic bacteria in the bacterial community. After caries formation, the local niche may be dominated by other bacteria, so that it is difficult to detect the *S. mutans* in the caries cavity. The “keystone” pathogen theory of periodontists holds that those bacteria that play a large role in the community but are of low abundance are considered to be the "keystone" members in the microbial ecology. Members of the microbiome with a low abundance may still be key species for complex community behavior [16]. *S. mutans* may be the "keystone" member of caries pathogens.

Some scholars have found that *S. mutans* can be detected in the caries, such as in the results of the study by Aas et al*.* using 16S rRNA sequencing to detect dental plaque. Aas et al*.* [17] found that *S. mutans* could not be detected in the plaque from healthy enamel and white plaque (demineralized surface of enamel) but could be found in most of the deep dentin plaque in caries cavity lesions. Research sample sources, sampling sites, sampling methods and research methods vary greatly, resulting in different abundances and detection rates of *S. mutans* in different studies. In this study, through qPCR detection, we found that the relative abundance of *S. mutans* in the saliva of children with early caries was significantly higher than that of children without caries, which was consistent with the previous results based on qPCR detection [18].

However, an increasing amount of evidence supports the idea that *S. mutans* is not the single causative factor of caries [19, 20]. In addition to *S. mutans,* bacteria of other genera, including *Lactobacillus, Actinomycetes, Bifidobacterium, Veronococcus, Propionibacterium,* etc., are believed to be involved in the occurrence and development of caries [17, 21]. Some bacteria, such as *Veronococcus,* have been found to be maintained in a high relative abundance at all stages of caries sites [17], and *Veronococcus* can participate in acid production at high glucose levels [22]. Interestingly, *Veronococcus* bacteria can synergize with *S. mutans,* and the coculture of *Veillonella alcalescens* and *S. mutans* can produce more acid than any single bacteria [23] suggest that both may play key roles in cariogenic disorders in the microbial community. Although this study did not find other microorganisms that might play a synergistic role with *S. mutans,* we still believe that dental caries is a multipathogen disease, and some core pathogens, including *S. mutans,* play a prominent role in the occurrence and development of dental caries.

Our hypothesis is based on the "core-pathogen complex" hypothesis. The oral cavity is home to hundreds of bacterial species, most of which are symbiotic and need to maintain balance with the oral ecosystem. Dental caries are caused or maintained by resident or temporary microorganisms, which are usually small in number and do not cause disease, but infection can occur in certain circumstances [24]. Some of the bacteria in the microbiome are the core pathogenic bacteria, which play key roles, while the rest are ordinary members or transient bacteria that do not play a key role in the microbiome. When all or some of the core pathogenic bacteria in the microbiome act together, the whole microbiome structure can be disturbed and progress towards the pathogenic state. Takahashi et al. [25] reported that the cariogenic microecosystem is a microenvironment that changes from a dynamic stability stage (maintaining dynamic stability on the tooth surface) to an acidogenic stage (increased proportion of acid-producing bacteria) and finally to an aciduric stage (promoting caries formation). We hypothesized that *S. mutans,* together with other core pathogens, may be the key bacteria for regulating the microecological balance of caries. Yang et al. [26] analyzed the saliva samples of caries-free and carious participants and found that it was difficult to generalize the caries-related "core microbiome" from the taxa level, but that the caries group had "core functional genes" at the gene level. Josh et al. [27] supported the idea that caries is a metabolic disorder of the microbial community rather than a change in species abundance. Due to the presence of homologous genes and horizontal gene transfer, different species of microorganisms may play similar functions in the host, so it is necessary to simultaneously analyze the species and genetic composition of caries-related microorganisms. Bacterial traits related to cariogenicity (acid production and acid tolerance) does not exclusively belong to several cariogenic bacteria but actually observed in a wide variety of species. Lactate dehydrogenase (LDH) plays as a key enzyme in bacterial extracellular organic acids production. F-ATPases, by pumping protons out of cells, function to maintain internal acid–base equilibrium. We used a qPCR method to quantitatively identify the *ldh* and *F-ATPase* genes, and the expression of *ldh* and *F-ATPase* genes reflect the overall capability of acid production and acid tolerance [28]. However, no significant differences in the functional genes were found between the two groups, which may be caused by the expression levels of the bacterial genes were more important than the presence of the functional genes, and thus the detection of the metatranscriptome may be more important.

The qPCR quantitative method can only be used to quantify a limited number of microbial species of interest in the community. Research on a single or limited species of bacteria can only be carried out selectively, which often fails to give a more comprehensive description of the overall community structure. High-throughput sequencing of the bacterial *16S rRNA* gene fragment amplified by PCR (*16S rRNA* gene sequencing) based on a nonculture method is a popular method for the study of the bacterial community, providing a simple and easy method for the species composition and structure of the entire bacterial community. However, at present, this approach is faced with several problems, such as the short lengths of the second-generation sequencing of the DNA fragment (which has insufficient analytic power for species), PCR amplification bias and chimera formation, too many variables leading to false positive statistical results, false negatives after multiple test corrections, and unknown sequences that do not match any known database. We did not find significant differences between the two groups with the results of *16S rRNA* gene sequencing, which may be caused by the limited accuracy of fragment sequencing analysis of the *16S rRNA* gene. The advantage of the "shotgun" metagenomic research method is that it can determine not only the species composition and relative abundance of the microbial community but also the microbial gene content and functional gene diversity. Moreover, the "shotgun" metagenomic method can obtain information at the bacterial strain level with a sufficient sequencing depth [29]. In this study, the *ldh* gene and the *F-ATPase* gene of bacteria that were related to caries formation were chosen for qPCR, and no differences were found. The differences in the genes need to be further studied through a shotgun metagenomic study with a large number of samples. However, from a single cross-sectional analysis and limited amount of samples, this study cannot determine whether variation across individuals represents a pattern of microbe interacts with human host. Further research should be undertaken to clarify the mechanisms that the interrelationship between microbial members and the association between microbial dysbiosis and human diseases.

## Conclusion

*S. mutans*, together with other pathogens, may play a prominent role and act as "core microbes" in the occurrence and development of early childhood caries.

## Data Availability

The datasets used and/or analyzed during the current study are available from the corresponding author upon reasonable request.

## Abbreviations

ECC: Early childhood caries
DMFT: Decayed, missing, and filled teeth
WHO: World Health Organization
*S. mutans*: *Streptococcus mutans*
SIC: Significant caries index
DGGE: Denaturing gel gradient electrophoresis
HOMIM: Human oral microbe identification microarray
OTUs: Operational taxonomical units
*S. sorbrinus*: Streptococcus sorbrinus
*ldh* gene: Lactate dehydrogenase gene

## References

[1] Pitts N, Baez R, Diaz-Guillory C, Donly K, Alberto Feldens C, McGrath C, Phantumvanit P, Seow W, Sharkov N, Songpaisan Y (2019). Early childhood caries: IAPD Bangkok declaration. J Dent Child (Chic).

[2] Tinanoff N, Baez R, Diaz Guillory C, Donly K, Feldens C, McGrath C, Phantumvanit P, Pitts N, Seow W, Sharkov N (2019). Early childhood caries epidemiology, aetiology, risk assessment, societal burden, management, education, and policy: Global perspective. Int J Pediatr Dent.

[3] Du M, Li Z, Jiang H, Wang X, Feng X, Hu Y, Lin H, Wang B, Si Y, Wang C (2018). Dental caries status and its associated factors among 3- to 5-year-old children in China: a national survey. Chin J Dent Res.

[4] Sanz M, Beighton D, Curtis M, Cury J, Dige I, Dommisch H, Ellwood R, Giacaman R, Herrera D, Herzberg M (2017). Role of microbial biofilms in the maintenance of oral health and in the development of dental caries and periodontal diseases. Consensus report of group 1 of the Joint EFP/ORCA workshop on the boundaries between caries and periodontal disease. J Clin Periodontol.

[5] Russell R (2009). Changing concepts in caries microbiology. Am J Dent.

[6] Jiang W, Jiang Y, Li C, Liang J (2011). Investigation of supragingival plaque microbiota in different caries status of Chinese preschool children by denaturing gradient gel electrophoresis. Microb Ecol.

[7] Ma C, Chen F, Zhang Y, Sun X, Tong P, Si Y, Zheng S (2015). Comparison of oral microbial profiles between children with severe early childhood caries and caries-free children using the human oral microbe identification microarray. PLoS ONE.

[8] Jiang W, Zhang J, Chen H (2013). Pyrosequencing analysis of oral microbiota in children with severe early childhood dental caries. Curr Microbiol.

[9] Marsh P (2006). Dental plaque as a biofilm and a microbial community—implications for health and disease. BMC Oral Health.

[10] Marsh P: Dental diseases—Are these examples of ecological catastrophes? *Int J Dent Hygiene* 2006:3–10; discussion 50–12. 10.1111/j.1601-5037.2006.00195.x 16965527

[11] Yang F, Zeng X, Ning K, Liu K, Lo C, Wang W, Chen J, Wang D, Huang R, Chang X (2012). Saliva microbiomes distinguish caries-active from healthy human populations. ISME J.

[12] A framework for human microbiome research. *Nature* 2012, 486(7402):215–221. 10.1038/nature11209 PMC3377744 22699610

[13] Loesche W (1986). Role of streptococcus mutans in human dental decay. Microbiol Rev.

[14] Belda-Ferre P, Alcaraz L, Cabrera-Rubio R, Romero H, Simón-Soro A, Pignatelli M, Mira A (2012). The oral metagenome in health and disease. ISME J.

[15] Xiao C, Ran S, Huang Z, Liang J (2016). Bacterial diversity and community structure of supragingival plaques in adults with dental health or caries revealed by 16S pyrosequencing. Front Microbiol.

[16] Hajishengallis G, Darveau R, Curtis M (2012). The keystone-pathogen hypothesis. Nat Rev Microbiol.

[17] Aas J, Griffen A, Dardis S, Lee A, Olsen I, Dewhirst F, Leys E, Paster B (2008). Bacteria of dental caries in primary and permanent teeth in children and young adults. J Clin Microbiol.

[18] Mitrakul K, Chanvitan S, Jeamset A, Vongsawan K (2017). Quantitative analysis of *S. mutans*, Lactobacillus and Bifidobacterium found in initial and mature plaques in Thai children with early childhood caries. Eur Arch Paediat Dent.

[19] Kleinberg I (2002). A mixed-bacteria ecological approach to understanding the role of the oral bacteria in dental caries causation: an alternative to Streptococcus mutans and the specific-plaque hypothesis. Crit Rev Oral Biol Med.

[20] Simón-Soro A, Mira A (2015). Solving the etiology of dental caries. Trends Microbiol.

[21] Becker M, Paster B, Leys E, Moeschberger M, Kenyon S, Galvin J, Boches S, Dewhirst F, Griffen A (2002). Molecular analysis of bacterial species associated with childhood caries. J Clin Microbiol.

[22] Bradshaw D, Marsh P (1998). Analysis of pH-driven disruption of oral microbial communities in vitro. Caries Res.

[23] Noorda W, Purdell-Lewis D, van Montfort A, Weerkamp A (1988). Monobacterial and mixed bacterial plaques of Streptococcus mutans and Veillonella alcalescens in an artificial mouth: development, metabolism, and effect on human dental enamel. Caries Res.

[24] Dahlén G (2006). Microbiological diagnostics in oral diseases. Acta Odontol Scand.

[25] Takahashi N, Nyvad B (2008). Caries ecology revisited: microbial dynamics and the caries process. Caries Res.

[26] Yang F, Ning K, Zeng X, Zhou Q, Su X, Yuan X (2016). Characterization of saliva microbiota's functional feature based on metagenomic sequencing. Springerplus.

[27] Espinoza J, Harkins D, Torralba M, Gomez A, Highlander S, Jones M, Leong P, Saffery R, Bockmann M, Kuelbs C (2018). Supragingival plaque microbiome ecology and functional potential in the context of health and disease. MBio.

[28] Li J, Wu T, Peng W, Zhu Y (2020). Effects of resveratrol on cariogenic virulence properties of Streptococcus mutans. BMC Microbiol.

[29] Dadi T, Renard B, Wieler L, Semmler T, Reinert K (2017). SLIMM: species level identification of microorganisms from metagenomes. PeerJ.

